# Patients’ perceptions of the meaning of good care in surgical care: a grounded theory study

**DOI:** 10.1186/s12912-016-0168-0

**Published:** 2016-08-03

**Authors:** Tünde Mako, Pernilla Svanäng, Kristofer Bjerså

**Affiliations:** 1 Department of Surgery, Linköping University Hospital, Linköping, Sweden; 2 Medical Faculty at Linköping University, Linköping, Sweden; 3 Division of Nursing Science, Faculty of Medicine and Health Sciences, Linköping University, Linköping, Sweden; 4 Department of Surgery, Clinical Sciences, Sahlgrenska Academy, University of Gothenburg, Gothenburg, Sweden

**Keywords:** Good care, Surgical care, Patient participation, Patient-centred care, Quality improvement, Evidence-based practice, Nurse

## Abstract

**Background:**

Patients in surgical care have reported a fear of being discharged prior to sufficient recovery and a lack of control of their situation. Establishing the patient-nurse relationship is essential in the context of the care. The Swedish National Board of Health and Welfare has established indicators for good care for comparison, evaluation and improvement of the quality of the health care system. These indicators are knowledge-based, appropriate, safe, effective and equal health care, as well as care within a reasonable time and patient-centred care. Current core competence in nursing education include quality improvement, patient-centred care, teamwork and collaboration, using evidence-based practice, safety and informatics. This study investigates patients’ perceptions of the meaning of good care in inpatient surgical care.

**Methods:**

Grounded theory according to Charmaz was chosen as the study design. Interviews were conducted with 13 patients from six surgical wards in the south of Sweden in 2014–2015.

**Results:**

The results showed that patients in surgical care perceived good care as being safe, as they were vulnerable and anxious. This could be achieved through accessible care, reliable care, caring attitudes and participating in one’s own care. Patient participation was achieved by information and education and the possibility to affect their care.

**Conclusion:**

Patients need safety to experience good care. Caring attitudes and patient participation can be attained through patient-centred care. Bedside handover can improve patients’ perceptions of accessible care and reliable care and can increase patient participation. Continuously maintaining competence and using evidence-based practice are needed to achieve reliable care.

## Background

Pressure on Swedish surgical care increases with the greater number of operations performed per year [1] while the number of hospital beds decreases [1, 2]. Over the past decade, the length of stay at hospital has decreased in all of the European Union countries; explanatory factors are a larger number of day surgeries and expansion of outpatient care and follow-up in primary care [1, 2].

Patients in surgical care have reported anxiety about being discharged before they are sufficiently recovered, not being ready to manage their own care and a lack of control of their situation [3–5]. There are positive experiences in surgical care when nurses give emotional support to patients that experience stress or anxiety [3]. A factor that increases patient satisfaction in surgical care has been found to be willingly given information by the nursing staff in layman terms about the disease [3]. Patients express an interest in being a part of the decision making process [6, 7] and having to fight for their autonomy is a source of suffering [6].

To clarify the fundamentals of care, Kitson et al. [8] analysed nursing theories and nursing research. They found it to consist of several dimensions and contexts, including safety, nutrition and elimination as well as mobility, rest and personal hygiene. Establishing the patient-nurse relationship is essential in the context of care, with the patients’ needs in focus, regardless of the diagnosis [8, 9]. According to cancer patients, a good patient-nurse relationship where there are discussions about things that are not related to the care give meaning and a feeling of good care [7, 10]. Patients with long-term illnesses experience care-related suffering when nursing staff do not see them as a whole human but just a diagnosis or an object [6]. They feel mistreated when the staff do not listen to them and do not take their symptoms seriously [6, 11].

Lower rates of patient mortality and morbidity have been observed in hospitals where nurses report good quality of care [12]. Nurses should adopt and develop competence in quality improvement in nursing education programs according to the Quality and Safety Education for Nurses faculty and National Advisory Board (QSEN) in order to enhance the quality of care and patient safety [13]. Along with quality improvement, the competences defined by QSEN are patient-centred care, teamwork and collaboration, using evidence-based practice, safety to reduce harm, and informatics. International and national comparisons of nursing quality can be used to raise quality and the competence of nurses [14]. The National Board of Health and Welfare in Sweden has established indicators for good care for comparison, evaluation and improvement of the quality of the health care system [15]. These are knowledge-based, appropriate, safe, effective and equal health care, care given within a reasonable time and patient-centred care.

In summary, health care strives towards good quality care through quality improvement and patient safety. There is no previous research to our knowledge about how QSEN and indicators of good care correspond with patients’ perceptions of good care. Surgical care affects the patient physically and emotionally, which must be taken into consideration in ensuring good care in this context of care. Further research is thus needed to investigate the meaning of good care in surgical care.

## Methods

### Aim

The aim of this study was to investigate patients’ perceptions of the meaning of good care in inpatient surgical care.

### Design

Grounded theory was chosen as the methodological approach and design based on the assumptions and interpretations presented by Charmaz [16]. The aim of grounded theory is to produce a theory from qualitative data and analysis [16–18]. Grounded theory according to Charmaz is commonly used in nursing research as the focus lies in understanding social behaviours and processes in patient care [19]. Little is known about the meaning of good care according to patients in surgical care. Hence, grounded theory, with its inductive core, was considered the most appropriate method for this study.

### Setting and participants

A request to perform the study and recruit patients was sent to six surgical wards at two hospitals in Sweden. All heads of departments accepted the request. The researchers had no work related connection to the wards invited. Inclusion criteria were patients cared for in a surgical ward between a minimum of 3 days and a maximum of 30 days, being discharged from the ward less than 2 months previously, being able to speak and understand Swedish, and provision of verbal and written consent. Exclusion criteria were transfer to and discharge from another hospital or clinic, and cognitive impairment. Patients were informed about the study and invited to participate on the day of discharge through written information distributed by the nursing staff. Patients who were interested filled out a declaration of interest and sent it to the researchers for further information and inclusion in the study. All these patients were contacted by the researchers by phone. Twenty-four interest forms were received and 13 patients were included. The time lapse between discharge and the interview had to be less than 2 months. Among the patients who were not included, three stated a feeling of being too ill to participate, two did not have time and one declined owing to a lack of interest. Five were actively excluded due to data saturation. The participants were chosen in accordance with theoretical sampling [16]. Interested patients were included if their experiences or characteristics could fill the gaps that emerged in the initial codes in the data, as described in theoretical sampling [16]. The interviews were held consecutively as the interest forms were received from November 2014 to January 2015.

### Data collection

Data were collected in individually recorded face-to-face interviews in private environments. The informants could choose the interview location to be either in their homes or at the university. Among the 13 informants, one chose the university, where the authors prepared a private room. To begin with, the interviews were semi-structured with open-ended questions. As the interviews continued, the interview guide was adapted by adjusting the questions to follow the theoretical direction and to fill the gaps in the data to reach the study aim [16]. Two questions were included in the interview guide in all of the interviews:

- Can you describe a situation during your stay where you experienced good care?
- Can you describe a situation during your stay where you experienced poor care?

No changes were made to the interview guide after the tenth interview. Field notes were taken during the interviews but were not used further in the data analysis.

### Data analysis and saturation

The researchers transcribed all the records verbatim, unidentified and numbered. Rules for transcription were set prior to data collection. Laughs and pauses were noted in the transcriptions. The analysis was made according to Charmaz [16]. The analysis starts with an initial coding, which means separating segments of the data and labeling them with simple and precise words. These codes are tentative and the researchers should maintain an open mind during the analysis, stay close to the data while coding and constantly compare data with data. The initial coding includes looking for implicit actions and meanings, crystallising the significance in the data and identifying gaps in the data. In focused coding, the initial codes are used to sort and analyse the data and to identify a theoretical direction. The codes that reveal patterns and best accounted for the data serve as focused codes. Here, focused coding was done after each interview and then compared with previous initial codes and focused codes. In the process of theory building, the focused codes were analysed and theoretical categories were created. These categories gave a theoretical direction to the interviews. An example of the analysis process is shown in Table 1.

**Table 1:** Example of the analysis process

Focused codes	Initial codes	Transcript
Caring attitudes—to be acknowledged	Be well cared for	Researcher: “We can start with you describing a situation where you thought that this was good or it was made well”
	Be listened to	Informant: “Well it was that I was really well cared for and listened to about my fears. I really wanted as much as I could have so that I would not be aware of it. They understood me really well and it wasn’t, no matter who I spoke to they were understanding, I was met with understanding, to give me sedatives and stuff. And they continuously asked about how you felt, you were not supposed to be in pain, and the importance of that it is harder for the body to be in pain than to take pain killers, that felt safe I think, you didn’t have to lie there and feel like a wuss, I could just ask for more. It felt really positive.”
Patient participation—be able to affect the care	To be able to affect the care	
Caring attitudes—to be acknowledged	Be met with understanding	
	Fears are taken seriously	
Caring attitudes—to care	Be asked about how you felt	
Safety	Safe not to be in pain	
Accessible care	Be able to ask for more	

The gathering of data continued as long as there was no saturation. No new dimensions or properties emerged in the categories in the analysis of the tenth interview. Three more interviews were held in which no further or deeper data were obtained but only confirmations of previous findings. This was interpreted as saturation. When the categories and focused codes were saturated, they were compared and analysed to identify their interactions and relationships to create a constructivist theory [16].

### Rigour

To minimise the risk of recall bias among the informants, a maximum time lapse of 2 months was set between discharge and the interviews. The first and second authors (TM & PS) participated in the first three interviews to give each other feedback. The remaining ten were held individually to lessen the informants’ discomfort. Because the interview guide changed during the data collection process, the structure of the interviews varied; thus the individual variations of the researchers were not considered to have affected the results. The first transcription was done independently by the first two authors and then compared. The data collection and analysis aimed to reach saturation in the categories and to find recurring concepts. All of the recorded interviews, transcriptions and analyses were saved to secure an audio trail. The data collection and analysis were made consistently according to the method and explained above in detail, which strengthens the credibility of the study [20, 21]. The third author ensured triangulation by comparing the transcripts and codes with the findings.

The researchers are clinical surgical nursing care specialists working at a general surgical ward and thus have knowledge and experience of giving nursing care to patients undergoing surgery or conservative treatments for conditions in this surgical specialty. Any preconceptions or prejudices of the researchers were discussed before data collection started and during analysis to eliminate the risk of bias. An open mind during the initial coding and being strict with the transcriptions prevent the results from being affected by the preconceptions of the researchers [16].

### Ethics

The study was conducted as part of a Master’s thesis project in surgical nursing, advanced nursing science, and ethical considerations were monitored and approved by Linköping University in accordance with Swedish law on ethics in research [22]. Approval to contact and recruit patients and to conduct interviews was obtained from the department head at each of the six surgical wards. All participation in this study in terms of taking part in the interviews was voluntary, and all data were collected and analysed confidentially. Information was given both in writing and verbally, and written consent to participate was collected from all participants before the interview was held, in accordance with the Declaration of Helsinki and its amendments [23].

## Results

Thirteen of the 24 patients who returned the declaration of interest were included in the study, and these interviews constituted the basis of the analysis process. Among the patients included, six had been cared for at an urology ward, three at an orthopedic ward, two at a thorax ward and two at a general surgical ward. Seven of the informants were admitted from the emergency department and six were elective patients. The informants who did not undergo surgery received conservative treatment during the hospital stay. Informant demographics are presented in Table 2.

**Table 2:** Informant demographics (*n* = 13)

Length of hospital stay in days (median; min-max)	8 (3–15)
Age in years (median; min-max)	65 (54–84)
Gender (women/men)	8/5
Length of interviews in minutes (median; min-max)	30 (15–59)
Days between discharge and interview (median; min-max)	11 (5–22)
Informants undergoing surgery during hospital stay (*n*)	9

**Fig. 1:**
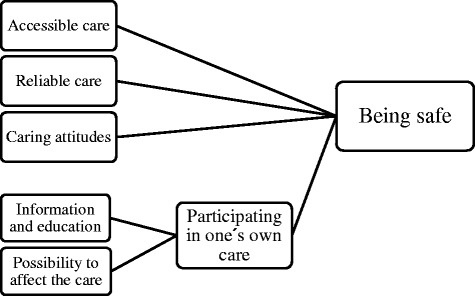
Model of good care in surgical care

The grounded theory process, where each interview is followed by an analysis, provided insight into the meaning of good care according to the patients cared for at surgical wards. The informants experienced being in a vulnerable position, which made them nervous and anxious. To have a perception of good care, the informants needed safety. Factors contributing to safety were accessible care, reliable care and caring attitudes among the staff. To be safe, the informants also needed information, education, and the ability to affect their care, enabling the informants to participate in their own care. The informants rarely distinguished between the different health care professions in the interviews. Rather, they mainly referred to “staff” in general, meaning a summary of all health care professionals. The model of good care that emerged from these findings is shown in Fig. 1.

### Accessible care

Accessible care was found to be a factor that contributed to good care, in reference to the informants receiving the help they needed when they asked for it and to the staff confirming their unspoken needs. A contribution to the experience of good care was fast help when needed, when the patients used the call bell. Fast assessment and management of current problems increased feelings of safety. To feel welcome and to get a bed at arrival to the ward contributed to the perception of accessible care.

Staff with sufficient time for the patients’ needs and who did not show stress increased the perception of accessible care, and hence the experience of good care. If the staff did not have time to help the patients but explained why, and said when they would come back, the informants still felt satisfied.

“We were always attended to and I know there were peaks [about staff workload] sometimes, they had a lot to do, I could see that, sometimes they had more time, but we were always, there was always someone there” Informant 11

The informants expressed the importance of knowing which member of the staff was responsible for their care in order to know who to ask for help. They appreciated having the staff nearby and being able to get their attention easily. Staff who came by the patients’ beds, checked on them a few extra times and offered help increased the feeling of accessibility.

“They rushed by and sure they were hurried and short-staffed and they looked away like ‘we do not see you’ and you are calling for attention and stuff … I would have wanted more presence “Informant 13

### Reliable care

The informants said it was important to be able to rely on staff doing their job correctly. Competent and professional staff increased the informants’ feelings of safety and lessened their anxiety, which promoted the experience of good care. Going through surgery at a specialized clinic where the staff had experience in the surgical procedure and in the postoperative phase made the informants feel safe. Staff that were easy to talk to and were engaged in the care increased the feeling of reliability and good care.

“Well it was mainly the way they were towards me but I mean they were all very competent and I thought that they supported each other” Informant 11

A threat to good care was what the informants perceived as inconsistency, as staff gave different answers or directions to the informants. This made them question the knowledge and competence of the staff. Inconsistency made them wonder whether they had gotten the correct care and whether the care was different depending on which staff member was responsible. When the staff were not able to make decisions about the care, the informants felt that the staff’s competence was inadequate. These situations made them feel that they could not rely on the care.

“But they [the staff] have different opinions about things. It was something with the drip ‘No he is not going to have any more drip’ and then the afternoon staff came and gave me a drip” Informant 3

The informants had diverse perceptions about having different physicians who made the daily rounds. Changing physicians from day to day could create the feeling of being forgotten and not having a physician who was responsible for them, which threatened the experience of receiving good care. Continuously meeting the same physician increased the feeling of safety. Some of the informants felt that they could trust the competence of an older physician more than of a younger one.

### Caring attitudes

The attitude of the staff towards the informants was a factor that affected the experience of good care. Caring attitudes were perceived as the staff being friendly and nice, using humour and being positive and optimistic. The informants wanted to feel taken care of, and they appreciated the staff making time for them, just to sit and talk for a few minutes, for no other reason than just to talk. Even a short meeting where the staff did not show stress was valuable for the informants. They expressed the importance of an easygoing relationship with the staff. They could talk about everyday or personal things and be on the same level, not feeling inferior to the staff. It was important not to feel like simply an object or a diagnosis, but to be perceived as a whole person.

“For me, it lifts me to be more positive and happy and just, really it is just small things that I need. When they come in and just say something nice. You somehow feel that you’re not just a social security number but… they just come in and say something, yes, that feels good, really good, for me.” Informant 3

It was important for the informants to feel that their experiences and opinions were significant, and that they were acknowledged and respected by the staff. Situations in which the staff avoided talking about the informants’ anxiety or fears or did not see things from the patients’ perspectives diminished the perception of good care. There were staff that only did what they were supposed to do and did not attempt to have a human relationship with the informants or did not try to be attentive. This was stated to be a threat to the experience of good care.

“You can tell the difference between staff, there are those who come in and can talk to people. And then there are those who do things and check on you but have a hard time talking, but we are different you know, some people can read others and get on the same level and talk for a while, but for some people it is uncomfortable” Informant 1

### Information and education

The informants needed information in order to be able to participate in their own care and to be safe. They wanted to know what had happened during their hospital stay, test results, planned interventions and follow-up. Those who had surgery wanted to know how they might feel afterwards, the expected post-operative phase and the severity of the operation. Staff that explained what they did and why the informants had to undertake strenuous activities made them motivated and helped them in their recovery.

“Because you lie there and you wonder and wonder what are they saying, how is it, what is up with me, what are they going to do and so on, yes to the smallest detail you should be involved” Informant 9

“Yeah they talked all the time about what they did and why they did it, and I thought that I learned about that as well” Informant 10

The informants wanted adapted information in different forms: verbal, written and visual information, separately or combined. Some of the informants felt safe when a relative or spouse took part in the information as well. Because of different thoughts about how detailed information they wanted, the informants thought the staff should adapt it depending on the particular patient. Information that contributed to the perception of good care was given at the right time and was easy to understand. The information should be delivered in an honest way.

“Now afterwards I have a few things, like what did they do and how did they do the surgery and stuff like that, and they probably told me right after the surgery but at that time I wasn’t receptive for anything” Informant 13

It was important that the staff were open to giving the informants information and that they could ask questions and be able to participate in their care. Many of them wished for information to be given to them without having to ask for it. Information given by different professions was valuable depending on the content of the information.

The informants felt safe when they knew how to handle their present and future needs. For this, they needed education about new problems and conditions, and individually adapted information and motivation. Independence was important as it increased the informants’ confidence and made them feel ready to go home. Receiving information about how to contact health care after discharge increased the informants’ feelings of safety.

“Before surgery I was there and got information about what it [the stoma] was all about, what to choose between, and before discharge they were there and showed me and I changed it myself and they saw how I did it” Informant 4

“She did everything and said ‘this is how you do it’ and explained everything she did with the catheter the first and the second time. It went well. And then I said ‘now it’s fine, I can do it myself’ and I did” Informant 8

### Possibility to affect the care

Staff that enabled informants to affect their own care and participate in decisions and staff that listened to the informants’ preferences contributed to the perception of good care. Decisions could include choosing between different food alternatives, having an influence on the day of discharge and being allowed to decide what activities of daily living they could handle. Negotiable staff that gave alternatives in treatments and interventions contributed to the perception of good care.

“What was really important was that they actually listened to what I wanted or thought, I really think they did that” Informant 4

“And it was really good when I thought that I didn’t want to have a urinary catheter and that the staff was negotiable and said ‘we can do a bladder scan instead and look how much urine is left’” Informant 6

### Core results—Being safe

The meaning of good care in surgical care is the process of becoming safe. This is achieved by accessible care, reliable care and caring attitudes, with simultaneously matched attributes such as welcoming patient participation in the care through information and education and giving the patient the possibility to affect the care. All of these aspects are closely related and affect each other. Good care cannot be accomplished by these factors separately as they are all essential in the overall process of being safe, and hence in experiencing good care as a patient in the surgical context.

## Discussion

The results show that good care consists of feelings of safety. These results concerning safety correlate with the fundamentals of care including a patient-nurse relationship, where safety is a major part [8].

The informants felt safe when the staff were accessible and could quickly give assistance. Surgical patients can feel secluded when moving from the postoperative unit to the ward [4], which according to these findings can be aided by visible and accessible staff. Patients felt safer when the staff checked on them often, as it increased their trust in being regularly assessed and receiving correct care. Further, we found that accessible care included staff giving time, offering help without patients asking for it and not showing stress in the patient encounter, which is in line with findings in a study by Vaismoradi et al. [24]. Nurses and surgeons report time-consuming administrative work and being interrupted by telephone calls or colleagues to be obstacles to achieving good care, as they reduce time with the patients and increase feelings of stress [25]. Support after discharge is another kind of accessibility that has been shown to be important for patients with a new stoma, which reinforces their autonomy [26]. Accessible care as a predictor of good care can be put in contrast to a study by Joffe et al., where patients’ experiences of accessible staff were associated with a willingness to recommend the hospital [27].

It is perceived by nurses that difficulties in establishing trust and confidence in the care are a threat to good care [28]. In this study, trust and reliable care were essential parts of good care. Reliable care was communicated by the staff via competence, knowledge and engagement in the care. According to Vaismoradi et al., patients’ feelings of safety are threatened when nurses lack the knowledge to give correct care in terms of the patients’ needs and when they make nursing errors [24]. This coincides with knowledge-based care as an indicator of good care [15]. The QSEN emphasizes the importance of learning how to develop health care through evidence-based practice to secure the quality of the care [13]. Organisational learning and continuous improvement work can reduce the risks of adverse events [29]. Successfully implementing evidence-based practice requires managers to make priorities and award implementation of evidence-based practice, adequate time and a low staff turnover [30].

Caring attitudes among the staff, as described in this study, were kindness and optimism. The informants valued a good relationship with the staff and not being inferior to the staff. A satisfying relationship between the nurse and the patient can lead to a higher capacity for self-care [31], and a high self-care capacity can strengthen patients’ autonomy and their possibility to make decisions about their care [32]. Respect and seeing the patient as a person are important parts of patient-centred care and lead to greater trust and understanding between the patient and the staff [33] and greater patient satisfaction [34]. Both the QSEN and the Swedish National Board of Health and Welfare describe patient-centred care as important components of good quality care. Person-centred care is another term that highlights the person behind the diagnosis instead of the patient as an object or a disease [35, 36]. The person should be treated as an equal and active partner in his or her own care, and as a person with a will, needs and feelings [35].

In order to be able to participate in their own care, the informants in this study needed adapted information and education. Having to wait for information or for getting a diagnosis can lead to patients feeling less safe [24] and having severe anxiety and a fear for their life [37]. Educating and informing patients preoperatively about the expected recovery reduce the patients’ anxiety and lead to patients being more responsible for their own recovery process and feeling safe after surgery [26, 38–40]. Further, proper information about one’s own care, such as treatment and side effects, may increase patients’ management of their self-care needs [41]. This correlates to the findings of this study, where the informants expressed independence and confidence as a result of education.

Patient participation may be improved by a bedside handover where the information between the staff is given in the presence of the patient. The patients continuously get information about their own care and become a partner in the caring process [42]. With a bedside handover, the patient can be ensured that the on-going staff are adequately informed about his or her care, which could increase the patient’s trust in the staff. Patient input and participation can improve the outcome, increase patient satisfaction and give the patient an opportunity to give correct and relevant information to the nurses [42, 43]. Bedside handover may also increase feelings of accessible care as the staff introduces themselves and the patient knows who is responsible for the caregiving.

### Strengths and limitations

The foundation to this study was the work of QSEN with indicators for good care and fundamentals of care defined according to Kitson et al. [8, 9]. Inductive study design aimed to produce building blocks for a new theory, with the use of grounded theory according to Charmaz [16]. This was perceived as a strength as Charmaz’s constructivist grounded theory combine both the positivistic theoretical approach, i.e on a universal and generalizable level seeks to explain relationships between concepts or variables through empirical work, and the interpretive theoretical approach, i.e. to understand the reality of the subjects in their cultural and social context with the assumption that truth is only provisional. As there is little known about the meaning of good care according to patients in surgical care, this dualistic approach towards evidence of the meaning of good care was considered an appropriate method.

Although the sample was thoroughly described in order to claim transferability, given the fairly small number of participants in this qualitative study, transferability is difficult to achieve [20]. No external triangulation was made by an analyst not connected to the study or a participating informant. Last, the total number of patients informed about the study at time of discharge was not registered and is thus unknown.

### Implications for practice

The findings highlight the significance of patient-centred care and person-centred care, which mean patient participation and caring attitudes among the staff. Bedside handover is a tool to improve patient participation by continuously informing the patient. Improvement work and learning throughout the work life should be a natural part of the health care organization in continuously improving the quality of the health care. Skills and knowledge about improvement work ought to be taught during nursing or medical school to enhance the safety and quality of health care.

### Further research

The attitudes of the staff have a great influence on how patients perceive care. Hence, in order to gain further insight into what good care in surgical care really consists of, it could be relevant to identify specific caring interventions or behaviours that increase patient participation and patient-centred care. A patient-reported experience measure may be designed from these findings to investigate the extent to which good care is achieved. In addition, the area of recovery after surgery may very well be merged with research in good care in surgical care in order to meet the approaching demand of fast-track surgery and recovery after surgery.

## Conclusion

According to patients, good surgical care means being safe and is achieved through accessible care, reliable care, caring attitudes and patient participation through information, education and having the possibility to affect one’s care. This definition correlates well with indicators for good care given by the Swedish National Board of Health and Welfare as well as with competences by QSEN, namely knowledge-based care /evidence-based practice, safety, and patient-centred care.

## Abbreviations

KB, Kristofer Bjerså; PS, Pernilla Svanäng; TM, Tünde Mako; QSEN, The Quality and Safety Education for Nurses faculty and National Advisory Board
